# Vector competence of *Culex quinquefasciatus* from Santiago Island, Cape Verde, to West Nile Virus: exploring the potential effect of the vector native *Wolbachia*

**DOI:** 10.1186/s13071-024-06609-7

**Published:** 2024-12-23

**Authors:** Aires Januário Fernandes da Moura, Filipe Tomaz, Tiago Melo, Gonçalo Seixas, Carla A. Sousa, João Pinto

**Affiliations:** 1 Global Health and Tropical Medicine (GHTM), Associate Laboratory in Translation and Innovation Towards Global Health (LA-REAL), Instituto de Higiene e Medicina Tropical (IHMT), Universidade NOVA de Lisboa (UNL), Rua da Junqueira 100, 1349-008 Lisbon, Portugal; 2 Unidade de Ciências da Natureza da Vida e do Ambiente, Universidade Jean Piaget de Cabo Verde, Praia, Cape Verde

**Keywords:** *Culexquinquefasciatus*, West Nile virus, *Wolbachia*, Vector competence, Cape Verde

## Abstract

**Background:**

*Culex quinquefasciatus* plays a crucial role as a vector of West Nile virus (WNV). This mosquito species is widely distributed in Cape Verde, being found in all inhabited islands of the archipelago. However, no data are currently available on the susceptibility of the local mosquito population to WNV. This study aimed to assess the vector competence of *Cx. quinquefasciatus* mosquitoes from Santiago Island, Cape Verde, for WNV and to explore the potential impact of its native *Wolbachia* on virus transmission.

**Methods:**

*Wolbachia*-infected and uninfected *Cx. quinquefasciatus* female mosquitoes were exposed to WNV lineage 1 PT6.39 strain using a Hemotek membrane feeding system. Mosquito samples, including the body, legs, wings and saliva, were collected at days 7, 14 and 21 post-infection (dpi) to assess WNV infection through one-step quantitative real-time PCR (RT-qPCR).

**Results:**

*Culex quinquefasciatus* from Cape Verde exhibited high susceptibility to the tested strain of WNV. Also, treated females without their native *Wolbachia* exhibited significantly higher WNV load in their bodies and greater dissemination rate at 7 dpi than their wild-type counterparts carrying *Wolbachia*.

**Conclusions:**

The high susceptibility to WNV of *Cx. quinquefasciatus* from Cape Verde poses a potential risk for virus transmission in the archipelago. However, *Wolbachia* infection in this mosquito species seems to confer protection against WNV dissemination in the early stages of viral infection. Additional research is required to uncover the mechanisms driving this protection and its potential impact on WNV transmission.

**Graphical abstract:**

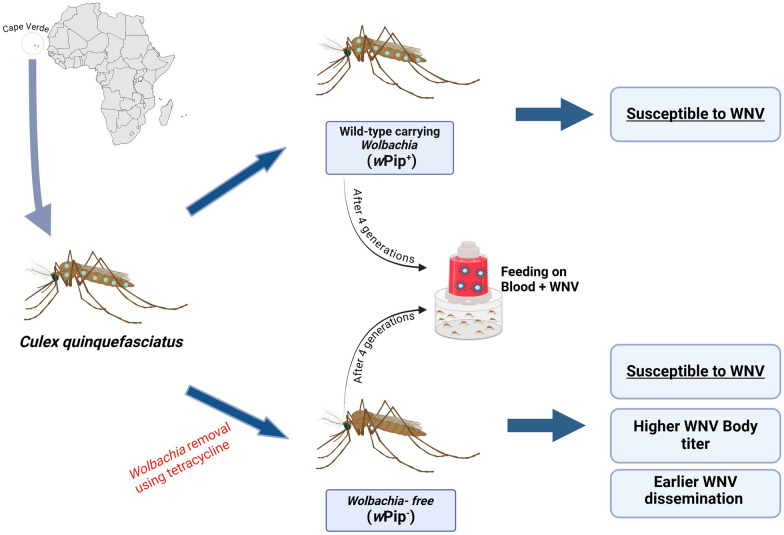

**Supplementary Information:**

The online version contains supplementary material available at 10.1186/s13071-024-06609-7.

## Background

West Nile virus (WNV) is a positive-sense RNA flavivirus belonging to the Flaviviridae family [1]. It was initially documented in Uganda in 1937, and since then it has spread across regions in Africa, Europe, Asia, North America, Australia and the Middle East, with multiple outbreaks [2]. Approximately 80% of individuals infected with WNV experience no symptoms, while the remaining 20% may display a broad spectrum of clinical manifestations, ranging from influenza-like symptoms to severe neurological complications and even death [3]. Severe forms of illness, which occur in up to 1% of infections, are primarily associated with older or immunocompromised individuals [4].

WNV is transmitted through the bites of mosquitoes, and it persists in the natural environment through a cycle involving vectors and birds [5]. Humans, horses and other mammalian hosts are designated as WNV dead-end hosts, as they can become infected but do not generate the virus loads necessary for the infection of a naïve mosquito [6].

Transmission of WNV has been associated with mosquitoes of different genera, including *Anopheles, Aedes, Culex, Culiseta, Mansonia and Ochlerotatus.* However, species of the *Culex pipiens* complex stand out as primary vectors of WNV due to their remarkable efficiency in transmitting the virus, widespread distribution, tendency to feed on birds and strong preference for domestic settings [7–9]. Among the species in this complex, *Cx. quinquefasciatus* is typically found in low-altitude regions between latitudes of 30° N and 30° S [10]. *Culex quinquefasciatus* is an important vector for WNV across the Americas, Africa and Asia, being accountable for the transmission of the virus in both rural and urban areas [11, 12].

*Culex quinquefasciatus* is naturally infected by *Wolbachia*, an obligate endosymbiotic bacterium found in approximately 66% of all insect species [13]. Although there is sufficient evidence that mosquitoes hosting transinfected *Wolbachia* strains exhibit reduced competence for arboviruses [14–16], there is limited information on the potential influence of *Wolbachia* infections on the transmission of arboviruses by their natural mosquito hosts. Studies on vector competence using *Cx. quinquefasciatus* mosquitoes treated to remove their native *Wolbachia* suggest that the endosymbiont can interfere with WNV dynamics in mosquito tissues [17, 18].

In Cape Verde, *Cx. quinquefasciatus* has a wide distribution, being present in all inhabited of the archipelago [19]. A previous study showed that *Cx. quinquefasciatus* populations from the islands harbor *Wolbachia* from the *w*Pip-III and *w*Pip-IV groups, with a total prevalence of 98.3% [20]. Although there are no recent records of WNV occurrence in Cape Verde, seroepidemiological surveys from the 1980s detected the circulation of the virus in humans, possibly introduced into the country by migratory birds [21]. Nevertheless, to date there has been no investigation aimed at determining the vector competence of Cape Verdian *Cx. quinquefasciatus* for WNV or the influence of circulating *Wolbachia* strains on the outcome of WNV infection.

The aim of the study reported here was to fill these gaps in current knowledge by testing the *Cx. quinquefasciatus* population from Santiago Island.

## Methods

### *Culex quinquefasciatus* field collection and colony establishment

Immature mosquitoes were collected in all Santiago Island municipalities, Cape Verde (15°07′48.0ʺ N, 23°31′48.0ʺ W) between August and October 2022. *Culex* sp. larvae were collected using standard sampling techniques with dippers and pipettes [22]. A subsample of collected larvae were morphologically identified to species complex using the identification key of Ribeiro and Ramos [23]; the remaining larvae were reared to adults in trays filled with dechlorinated tap water and fed with fish food (TetraMin; Tetra Werke, Melle, Germany). Adult mosquitoes were maintained at 28 °C and 70–80% relative humidity under a 12:12-h light:dark cycle, with access to a sucrose 10% solution ad libitum. One-week-old females were blood-fed on human blood using an artificial blood-feeding protocol described in Siria et al. [24] and following the Standard Operational Procedures adopted by the Instituto Nacional de Saúde Pública of Cape Verde. An oviposition container with water was placed inside the cage 4 to 5 days after blood-feeding.

The resulting F1 generation egg rafts were transported to the In Vivo Arthropod Security Facility (VIASEF) of the Institute of Hygiene and Tropical Medicine (IHMT), Portugal, where all experiments with WNV were conducted under biosafety level 3 (BSL3) conditions.

### *Wolbachia* detection and treatment with tetracycline

At VIASEF, *Cx. quinquefasciatus* egg rafts were reared to adults as described in the previous section. The resulting adult females were blood-fed with human blood on an artificial membrane feeder (Hemotek®, Blackburn, UK) and allowed to oviposit eggs to produce an F2 generation.

A subsample of 200 non-blood-fed F1 females was used for molecular confirmation of species and screening of *Wolbachia*. DNA was extracted individually from each specimen using 2% cetyltrimethylammonium bromide (CTAB), as previously described by Weeks et al. [25], and molecular identification was performed by amplifying species-specific gene fragments from the acetylcholinesterase-2 gene [26] and cytochrome* c* oxidase subunit 1 (COI) gene [27], using primers described in Additional file 1 Table S1. The acetylcholinesterase-2 PCR product was analyzed by gel electrophoresis, and the COI product underwent purification and DNA sequencing. The resulting sequences were queried in the Barcode of Life Data Systems (BOLD Systems) (https://www.boldsystems.org). A sequence was assigned to a species when it showed at least 98% similarity with the reference sequence in BOLD. *Wolbachia* infection and prevalence were assessed in the same F1 individuals by conventional PCR using the 81F and 691R primers [28], which amplify a 610-bp segment of the *Wolbachia* surface protein gene (*wsp*). Amplification conditions were performed as previously described by da Moura et al. [20].

A subset of F2 mosquitoes was treated to remove *Wolbachia*. The treatment was performed using tetracycline hydrochloride (Selleck Biotechnology GmbH, Cologne, Germany) following the protocol of Shemshadian et al. [29] with modifications. Briefly, *Cx. quinquefasciatus* stage 1 larvae (L1) were placed in an aqueous tetracycline solution (50 µg/ml) until pupation. The resulting adults were placed in a cage and fed on a solution containing 10% sucrose and 50 µg/ml of tetracycline hydrochloride. Adults were blood-fed on an artificial membrane feeder (Hemotek®) and allowed to oviposit eggs. The treatment was repeated for one more generation.

A real-time PCR (RT-PCR) assay was developed to improve the sensitivity of *Wolbachia* detection and evaluate the effectiveness of the tetracycline treatment. The primers were designed using multiple alignments of *wsp* gene sequences available in the National Center for Biotechnology Information (NCBI) database (Additional file: Table S2). The specificity of the amplified region was evaluated with Primer-BLAST (www.ncbi.nlm.nih.gov/tools/primer-blast) to confirm that the target of interest was unique. The RT-PCR reaction was performed in a reaction volume containing 10 µl iTaq Universal SYBR® Green Supermix (Bio-Rad Laboratories, Hercules, CA, USA), 0.1 µM of forward and reverse primers, 2 µl of DNA and water to a final volume of 20 µl. The thermocycling conditions consisted of one cycle at 95 °C for 5 min, followed by 35 cycles at 95 °C for 5 s and 60 °C for 15 s. Melting curve analysis was performed at 65–95 °C, with 0.5 °C increments with 2–5 s for each step. The quantitative PCR (qPCR) was conducted using a CFX96 real-time PCR detection system (Bio-Rad Laboratories).

After *Wolbachia* clearance, the treatment was stopped, and the treated and untreated mosquitoes (henceforth referred to as wPip^−^ and wPip^+^, respectively) were reared for four generations (until F7) in separated cages and trays but in the same room, using the same water and food, under a consistent nutritional regimen to ensure uniformity of the microbiome between the two lines. The F7 generation from both lines was used in experimental infections with WNV.

Since *Wolbachia* density may change under laboratory settings, which may account for variation observed in antiviral protection [30, 31], we used the RT-PCR assay described above to evaluate whether there were significant changes in *Wolbachia* density between the wild-type F1 generation and the non-treated F7 generation (wPip^+^) used in the artificial infections. For this purpose, DNA was extracted from both generations of mosquitoes using three biological replicates, each containing a pool of five whole-body females (collected 5 days post pupal eclosion). Total *Wolbachia* density was analyzed by relative quantification of the *Wolbachia* surface protein (*wsp*) against the 18S ribosomal RNA (rRNA) reference gene of *Cx. quinquefasciatus*. Primers for the 18S rRNA gene were designed using *Cx. quinquefasciatus* reference sequences from VectorBase and the Primer-Blast tool (Additional file 1: Table S2). The PCR reaction was conducted in a reaction volume containing 10 µl iTaq Universal SYBR® Green Supermix (Bio-Rad Laboratories), 0.1 µM of *wsp* primers, 0.5 µM of 18S ribosomal primers, 2 µl of DNA template and water to reach a final volume of 20 µl. The thermocycling parameters included one cycle at 95 °C for 5 min, followed by 35 cycles at 95 °C for 5 s and 60 °C for 15 s. Melting curve was performed at 65–95 °C, with 0.5 °C increments with 2–5 s for each step.

### Production and quantification of viral stocks

Vector competence assays were conducted using the WNV PT6.39 strain (GeneBank accession number: AJ965630.2), which is a lineage 1 WNV strain isolated in Portugal in 2004 [32]. WNV was propagated using African Green monkey kidney cells (Vero E6) maintained in Dulbecco’s Modified Eagle Medium (DMEM; Gibco, Thermo Fisher Scientific, Waltham, MA, USA) supplemented with 2% fetal bovine serum (FBS; Sigma-Aldrich, St. Louis, MO, USA), 2 mM l-glutamine, 1% penicillin/streptomycin (Sigma-Aldrich) and amphotericin B (Selleck Biotechnology GmbH), in a multiplicity of infection of 0.1. The cells were incubated at 37 °C with 5% CO_2_ for 2–3 days and checked daily until a 50% cytopathic effect was observed. Portions of the supernatant were employed to infect a fresh batch of Vero cells. After two consecutive passages, the supernatant was transferred to 1-ml cryotubes and stored in a freezer at − 80 °C until further use.

WNV particles in viral stocks were quantified using a plaque assay following the protocols of Brien et al. [33] and McAuley and Beasley [34] with modifications. In summary, 12-well tissue culture plates were seeded with 1.5 × 10^5^ Vero cells/ml on the day preceding the assay using DMEM medium containing 10% FBS, 2 mM l-glutamine, 1% penicillin/streptomycin and amphotericin B. On the day of the assay, the wells were washed with 1× phosphate buffered saline (PBS), then inoculated with 200 µl of serially diluted viral stock and incubated for 1 h. Following the incubation, 1 ml of the semi-solid medium was added to the well, comprising a 1:1 mixture of 1.5% high-viscosity carboxymethyl cellulose (CMC; Sigma-Aldrich) and 2× DMEM with 4% FBS. The plate was then incubated for 3 days at 37 °C with 5% CO_2_. Subsequently, the plate was fixed using 10% buffered formaldehyde for 1 h, washed twice with a 1× PBS solution, and stained with 1% crystal violet to visualize the plaques.

The viral titer of the stocks was 7.6 log_10_ plaque-forming units (PFU)/ml.

### *Culex quinquefasciatus* artificial infection with WNV

The infective blood meal was prepared by mixing blood and viral stock produced in the previous step to a final titer of 7.0 log_10_ PFU/ml. An aliquot of the infectious blood meal was stored at − 80 °C for subsequent back-titration by plaque assay.

Mosquito females between 7 and 10 days old from both the wPip^−^ and wPip^+^ colonies were kept in groups of 100 individuals in paper cups and exposed to the infective blood meal using a Hemotek® membrane feeding system for 1 h. After feeding, engorged females were selected following sedation with CO_2_, transferred to new paper cups and maintained inside a climate chamber (Percival Scientific, Inc., Perry, IA, USA) maintained at 28 °C and 70% relative humidity RH, under 12:12-h light:dark cycle, with access to a 10% sucrose solution.

At days 7, 14 and 21 post-infection (dpi), 30 mosquitoes per group were anesthetized using CO_2,_; the legs and wings were then removed and saliva was collected by inserting the mosquito proboscis into pipette tips filled with 5 µl of DMEM with 10% FBS for 60 min. The collected saliva was then transferred to a microtube containing 200 µl of DMEM supplemented with 10% FBS. The dissected body and legs plus wings were stored separately in microtubes containing 300 µl of complete DMEM with 10% FBS, 1% amphotericin B and three 2-mm-diameter glass beads for subsequent homogenization during the RNA extraction. All samples were kept at − 80 °C until further use in the RNA extraction.

### RNA extraction and detection of WNV

For RNA extraction, the body, legs and wings were homogenized twice at 7500 rpm for 10 s in a Precellys Evolution Homogenizer (Bertin Technologies, Paris Region, France), and then centrifuged at 3000*g* for 15 min. The supernatant was collected and used in the RNA extraction. RNA was extracted from 100 µl of mosquito saliva or tissue supernatants using NZYol, according to the manufacturer’s protocol (Nzytech, Lisbon, Portugal). The RNA was further treated with Turbo DNase (Ambion, Austin, TX, USA) to remove genomic DNA and then stored at − 80 °C until further use.

WNV in each sample was detected and quantified by one-step RT-qPCR. The RT-qPCR was performed using the primers and probes described by Lanciotti et al. [35], which amplify a 70-bp fragment of the virus envelope gene (Additional file 1: Table S3). The RT-qPCR was performed in a reaction volume containing 10 µl of Xpert One-Step Fast Probe (GRiSP, Porto, Portugal), 0.4 µM of each primer, 0.1 µM of probe, 0.8 µl of RTase mix, 5 µl of RNA template (unit mass normalized) and water to a final volume of 20 µl. The thermocycling conditions consisted of complementary DNA (cDNA) synthesis at 50 °C for 20 min, one cycle at 95 °C for 5 min, 40 cycles at 95 °C for 5 s and 60 °C for 30 s. WNV copies per microliter were extrapolated from a standard curve generated in each reaction by serial dilutions of a synthetic oligonucleotide (gBlocks®; IDT Inc., Coralville, IA, USA) encompassing the 70-bp target region. The number of copies of WNV per milliliter of homogenized tissue and saliva was calculated by considering the RNA elution volume (30 µl) and the volume used for RNA extraction (100 µl).

Aliquots of saliva that tested positive for WNV in the RT-qPCR analysis were titrated on Vero cells to validate the infectivity of viral particles.

### Data analysis

The RT-qPCR results from each mosquito tissue and saliva were used to calculate the infection rate (percentage of WNV-positive bodies out of the total exposed mosquitoes), dissemination rate (percentage of virus-positive legs + wings out of the total number of positive mosquito bodies) and transmission efficiency (proportion of mosquitoes with positive saliva among the total number of fed mosquitoes). To detect potential differences in salivary gland infection barriers between wPip^+^ and wPip^−^ lines, we also calculated the transmission rate, which is defined as the percentage of mosquitoes with infected saliva out of those with disseminated infection.

Fisher's exact test was used to test for significant differences between wPip^+^ and wPip^−^vector competence indices at each day post-infection. After assessing the normality and homogeneity of the data variances, the non-parametric Mann–Whitney U-test was applied to compare the viral loads between the two groups.

Statistical analyses were performed using GraphPad Prism for Windows, version 10.1.2 (GraphPad Software, Boston, MA, USA) and IBM SPSS Statistics for Windows, version 29.0.1.0 (SPSS IBM Corp, Armonk, NY, USA).

## Results

### *Wolbachia* screening and efficacy of tetracycline treatment

**Fig. 1:**
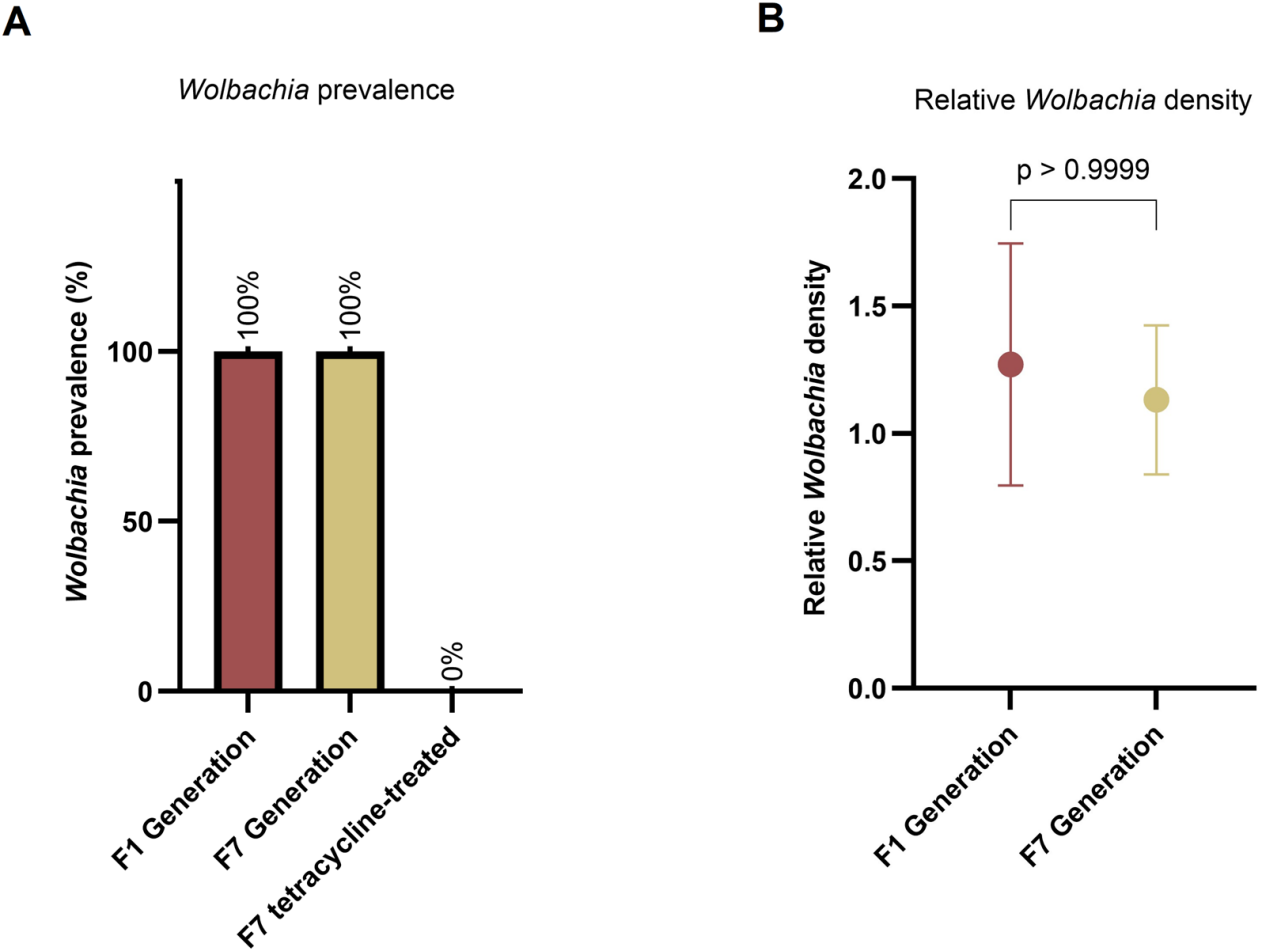
*Wolbachia* detection in *Culex quinquefasciatus* from Santiago Island, Cape Verde, based on the *Wolbachia* surface protein gene (*wsp*). **a** *Wolbachia* prevalence in F1 and F7 generations of wild-type mosquitoes, as well as in the F7 generation group treated with tetracycline (*n* = 200 per group), analyzed using conventional and real-time PCR. **b** *Wolbachia* density on a linear scale relative to the *Cx. quinquefasciatus* 18S ribosomal gene, as estimated by quantitative real-time PCR. The difference in *Wolbachia* density between the F1 and F7 generations was assessed using the Mann–Whitney test. Error bars represent 95% confidence intervals from three biological replicates, each containing DNA from a pool of 5 females

There was a 100% prevalence of *Wolbachia* in the F1 generation of *Cx. quinquefasciatu*s. Following tetracycline treatment, both conventional and RT-PCR results confirmed the successful elimination of *Wolbachia* in the treated mosquito group (wPip^−^) (Fig. 1).

Among the untreated group (wPip^+^), the evaluation of *Wolbachia* density by qPCR revealed no statistically significant differences between generations F1 and F7 (Mann-Whitney U-test, U_(6)_ = 4, Z = − 0.22, *P* > 0.9999) (Fig. 1).

### Vector competence

The WNV infection rates of *Wolbachia*-uninfected mosquitoes (wPip^−^) were 86.7% at 7 dpi, 83.3% at 14 dpi and 90.0% at 21 dpi. For mosquitoes harboring *Wolbachia* (wPip^+^), the infection rates were 76.7% at 7 dpi, 63.3% at 14 dpi and 93.3% at 21 dpi (Table 1). Although the infection rates at 7 and 14 dpi were higher for mosquitoes without *Wolbachia*, these differences were not significant (Fisher's exact tests, *P *= 0.506, OR = 1.98, 95% CI = 0.51–7.64, and *P* = 0.143, OR = 2.89, 95% CI = 0.86–9.74, respectively).

**Table 1:** Infection, dissemination, transmission rates and transmission efficiency for the population of* Culex quinquefasciatus* from Santiago Island exposed to West Nile virus PT6.39 strain

Days post-infection	Infection rate^a^ (%) [95% CI]			Dissemination rate^b^ (%) [95% CI]			Transmission rate^c^ (%) [95% CI]			Transmission efficiency^d^ (%) (95% CI)		
	wPip^−^	wPip^+^	*P-*value^e^	wPip^−^	wPip^+^	*P-*value^e^	wPip^−^	wPip^+^	*P *value^e^	wPip^−^	wPip^+^	*P *value^e^
7	26/30 (86.7) [69.7–95.3]	23/30 (76.7) [58.8–88.5]	0.506	13/26 (50.0) [32.1–67.9]	3/23 (13.0) [3.7–33.0]	0.007*	3/13 (23.1) [7.5–50.9]	0/3 (0.0) [0.0–61.8]	-	3/30 (10.0) [2.7–26.4]	0/30 (0.0) [0.0–13.5]	-
14	25/30 (83.3) [66.0–93.1]	19/30 (63.3) [45.5–79.2]	0.143	25/25 (100.0) [84.2–100.0]	16/19 (84.2) ]61.6–95.3]	0.073	13/25 (52.0) [33.5–70.0]	7/16 (43.8) [23.1–66.9]	0.751	13/30 (43.3) [27.4–60.8]	7/30 (23.3) [11.5–41.2]	0.170
21	27/30 (90.0) [73.6–97.3]	28/30 (93.3) [77.6–99.2]	1.000	26/27 (96.3) [80.2–99.9]	27/28 (96.4) ([0.8–99.9]	1.000	23/26 (88.5) [70.2–96.8]	18/27 (66.7) [47.7–81.5]	0.099	23/30 (76.7) [58.8–88.5]	18/30 (60.0) [42.3–75.4]	0.267

CI, Confidence interval (computed by the modified Wald method)

*Statistically significant difference between wPip^–^ mosquitoes (those treated with tetracycline hydrochloride to clear* Wolbachia*) and wPip^+^ mosquitoes (untreated mosquitoes)

^a^Infection rate is the percentage of West Nile virus-positive bodies out of the total number of exposed mosquitoes

^b^Dissemination rate is the percentage of virus-positive legs + wings out of the total number of positive mosquito bodies

^c^Transmission rate is the percentage of mosquitoes with infected saliva out of the number with disseminated infection

^d^ Transmission efficiency is the proportion of mosquitoes with positive saliva among the total number of fed mosquitoes

^e^*P*-value represents Fisher’s exact test to verify significant differences between wPip^–^ and wPip^+^ rates at each day post-infection

**Fig. 2:**
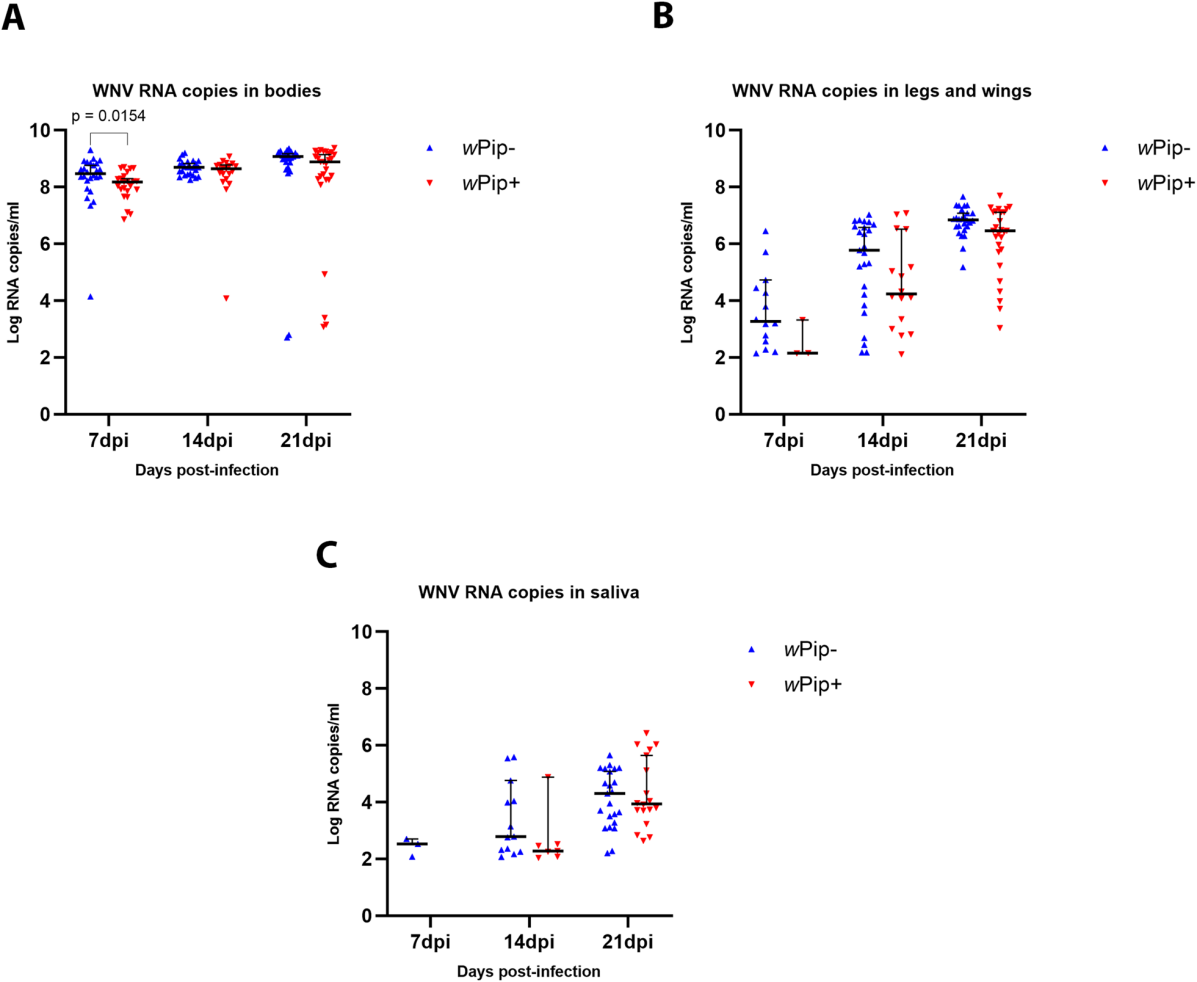
West Nile virus RNA copies in *Culex quinquefasciatus* body (**a**), legs + wings (**b**) and saliva samples (**c**) at different days post-infection for wPip^+^ and wPip^-^ colonies. At each time point, 30 mosquito bodies were screened for WNV. Error bars represent the median with 95% confidence intervals. Statistical significance at* P* < 0.05 was determined by the Mann–Whitney U-test at each day post-infection. dpi, Day post-infection; WNV, West Nile virus; wPip^−^/wPip^+^, mosquitoes treated with tetracycline hydrochloride to clear* Wolbachia*/untreated mosquitoes

The viral load in the mosquitoes’ bodies remained consistently elevated throughout the experiment (> 8 log_10_ copies/ml), regardless of the presence or absence of *Wolbachia*. However, at 7 dpi, we found that the number of WNV copies in mosquitoes lacking *Wolbachia* was significantly higher compared to those harboring *Wolbachia* (Mann-Whitney U-test, U_(49)_ = 179, Z = − 2.41, *P* = 0.0154) (Fig. 2a).

WNV dissemination rates in mosquitoes lacking *Wolbachia* surpassed those observed in mosquitoes harboring the endosymbiont (Table 1). Among mosquitoes carrying *Wolbachia*, rates of 13.0% at 7 dpi, 84.2% at 14 dpi and 96.4% at 21 dpi were recorded; in comparison, mosquitoes lacking *Wolbachia* exhibited dissemination rates of 50% at 7 dpi, 100% at 14 dpi, and 96.3% at 21 dpi. However, differences between the two groups were significant only at 7 dpi (Fisher’s exact test, *P* = 0.007, OR = 6.67, 95% CI = 1.59–28.04) (Table 1).

The quantity of viral RNA copies in legs plus wings increased in both groups as the extrinsic incubation period of the virus advanced. In these tissues, mosquitoes lacking *Wolbachia* exhibited higher titers than mosquitoes harboring *Wolbachia*, but the differences were not statistically significant (Mann–Whitney U-tests, U_(17)_ = 8.50, Z = − 1.58, *P* = 0.121 at 7 dpi; U_(41)_ = 149, Z = − 1.36, *P* = 0.179 for 14 dpi; and U_(53)_ = 241, Z = − 1.97,* P* = 0.05 at 21 dpi) (Fig. 2B).

Regarding transmission rates at 7 dpi, WNV was exclusively detected in the saliva of *Wolbachia*-free mosquitoes, with a transmission rate of 21.4%. At 14 dpi, mosquitoes harboring *Wolbachia* exhibited a transmission rate of 43.8%, compared to 52.0% recorded in mosquitoes lacking *Wolbachia*. At 21 dpi, the transmission rate for the *Wolbachia*-positive and *Wolbachia*-negative mosquitoes was 66.7% and 88.5%, respectively (Table 1). However, the differences in transmission rate between the two groups at 14 and 21 dpi were not significant (Fisher's exact tests, *P* = 0.751, OR = 1.39, 95% CI = 0.39–4.92, and *P *= 0.099, OR = 3.83, 95% CI = 0.90–16.26, respectively ).

The transmission efficiency for mosquitoes lacking *Wolbachia* was 10% at 7 dpi, and no viral transmission occurred in the wPip^+^ group during this incubation period, as previously noted. By 14 dpi, transmission efficiencies of 43.3% and 23.3% were recorded for the wPip^−^ and wPip^+^ groups, respectively. At 21 dpi, peak transmission efficiencies were observed in both groups, reaching 76.7% for the wPip^−^ group and 60% for the wPip^+^ (Table 1). Although higher transmission efficiencies were noted at 14 and 21 dpi for mosquitoes lacking *Wolbachia*, these differences were not significant (Fisher's exact tests, *P* = 0.170, OR = 2.51, 95% CI = 0.83–7.64 for 14 dpi, and *P* = 0.267, OR = 2.19, 95% CI = 0.72–6.70 at 21dpi).

WNV RNA copies in saliva increased with the extrinsic incubation period of the virus. At 14 and 21 dpi, mosquitoes without *Wolbachia* exhibited higher titers, although the differences were not statistically significant (Mann–Whitney U-tests, U_(20)_ = 27.5, Z = − 1.43, *P* = 0.157, and U_(41)_ = 225, Z = 0.47, *P* = 0.636, respectively) (Fig. 2c).

## Discussion

The results of this study showed that *Cx. quinquefasciatus* mosquitoes from Santiago are susceptible to the WNV strain tested during our investigation. To the best of our knowledge, this is the first assessment of the vector competence of a *Cx. quinquefasciatus* population from Cape Verde to WNV. The high transmission efficiency observed for the tested lineage 1 of WNV holds significant epidemiological importance for the archipelago, as it is the most prevalent lineage on the African continent, where it co-circulates concomitantly with lineages 2 and 8 [36].

Lutomiah et al. [37] tested the vector competence of *Cx. quinquefasciatus* from Kenya for WNV and found a transmission efficiency of 46% at 21 dpi. In Senegal, Fall et al. [11] documented a maximum transmission rate of 25% at 15 dpi for the local population of *Cx. quinquefasciatus*. In both of these African countries, WNV-infected *Cx. quinquefasciatus* has been detected in the field [36, 38], alongside the detection of virus in humans, birds and horses [3, 39]. These observations highlight the potential of *Cx. quinquefasciatus* for sustaining WNV transmission in Africa and the implications of our findings for the risk of WNV transmission in Cape Verde.

In other regions of the world, studies on the vector competence of *Cx. quinquefasciatus* for WNV have shown variable transmission rates. In Florida (USA), Richards et al. [40] documented low transmission rates for the local population of *Cx. quinquefasciatus*, with maximum values of 20% at 14 dpi. Goddard et al. [41] recorded maximum transmission efficiencies of 36–52% in California at 14 dpi. In China, Jiang et al. [42] reported a transmission efficiency of 37% at 14 dpi. In Europe, vector competence studies for WNV with the siblings species *Cx. pipiens* sensu stricto (*Cx. pipiens* s.s.) showed a maximum transmission efficiency of 21–33% at 32 dpi in Italy [9] and 40–53% at 21 dpi in Germany [43]. Overall, these values align with those obtained in the present study for Cape Verdean *Cx. quinquefasciatus.* Nonetheless, comparisons of WNV vector competence must be made with caution, as variations in transmission can be influenced by several factors, including the genetic variability of virus strains, incubation temperature, viral titer in the infected blood and mosquito populations studied [44–46]. These disparities underscore the need to standardize these parameters across studies to ensure comparability and consistency.

A recent study showed that *Cx. quinquefasciatus* from the island of Santiago prefers to feed on human blood, followed by chicken blood [47]. This mammophilic and ornithophilic tendency translates into a high potential for this mosquito to act as a bridge vector between avian and human hosts. In addition to this species, *Cx. pipiens* s.s. and hybrids between *Cx. pipiens* s.s. and *Cx. quinquefasciatus* are also present in Cape Verde [20, 48]. This may pose an increased risk for WNV transmission in the country since experimental infections have demonstrated a higher vector competence of hybrids compared to their parental species [49].

The lack of recent reports of WNV circulation in Cape Verde may reflect the absence of active virus surveillance. Cape Verde lies along the migration route of numerous African and European bird species [50–52], including the Eurasian collared dove (*Streptopelia decaocto*) and various species of herons and storks, all of which are well-known WNV reservoirs and could potentially initiate local transmission in the presence of competent vectors. Genetic surveillance studies suggest substantial WNV circulation between Europe and West Africa, consistent with the migratory flyways of Afro-Palaearctic birds, including movements to and from neighboring countries of Cape Verde [3, 53]. In this context, the introduction of WNV surveillance in migratory and wild birds in Cape Verde, as well as in mosquitoes and humans living near the nesting areas, with the aim to confirm the current status of WNV circulation in the country, would be an important initiative.

Our results showed that the treated *Cx. quinquefasciatus* (wPip^−^; *Wolbachia* free) exhibited a higher WNV dissemination rate than their wild-type counterparts, resulting in an earlier virus presence in the saliva of the former. Glaser and Meola [17] documented similar results, showing that *Cx. quinquefasciatus* lacking *Wolbachia* had a significantly higher viral dissemination rate than those harboring native *Wolbachia.* Alomar et al. [18] showed that the absence of *Wolbachia* in *Cx. quinquefasciatus* infected with WNV produces a higher viral load in conditions of low competition stress. In *Aedes albopictus*, native *Wolbachia* also appears to contribute to differences in arbovirus dissemination in mosquito tissues. Mousson et al. [54] showed that *Ae. albopictus* naturally super-infected with the *Wolbachia* strains *w*AlbA and *w*AlbB exhibited reduced dengue virus (DENV) dissemination to the wings compared to mosquitoes without *Wolbachia*. In another study with *Aedes albopictus*, Tsai et al. [31] found that the presence of native *Wolbachia* was linked to reduced DENV titers in mosquito tissues.

Although our findings indicate that native *Wolbachia* confers an antiviral phenotype during WNV infection, other studies have reported conflicting results. Leggewie et al. [55] found no association between *Wolbachia* and WNV infection in experimentally exposed *Cx. pipiens* s.s. from Germany. Micieli and Glaser [30] observed a protective effect of *Wolbachia* against WNV in a long-established laboratory colony of *Cx. quinquefasciatus*, but reported that a similar effect was absent in new colonies of the same mosquito species. More recently, however, Novakova et al. [56] demonstrated that an increased prevalence of WNV in field-caught *Culex pipiens* sensu lato from Canada was correlated with a reduced level of native *Wolbachia*, suggesting that the endosymbiont can induce protection in natural settings.

*Wolbachia* can be found in different tissues of the *Cx. pipiens*, including the midgut [57]. The lower dissemination rate we observed in *Cx. quinquefasciatus* harboring *Wolbachia* suggests that the endosymbiont may potentially integrate either the midgut infection barrier or the midgut escape barrier, delaying the virus dissemination to the legs, wings and saliva. Our data revealed that WNV load in mosquito bodies lacking *Wolbachia* was significantly higher than that in those harboring *Wolbachia*, and this disparity may have contributed to the observed increase in viral dissemination in *Wolbachia-*free mosquitoes. Several studies have demonstrated that viral dissemination within mosquitoes requires the virus load to reach a threshold in the insect's midgut [58, 59], implying that mosquitoes with midgut-to-hemocoel dissemination must have a higher viral load in their body than those without dissemination. Furthermore, it has been shown that manipulation of the RNA interference (RNAi) pathway in the midgut to either enhance or reduce virus replication resulted in a corresponding decrease or increase in viral disseminated infection [60]. These findings suggest that virus replication in the midgut significantly impacts dissemination, and our data indicate that native *Wolbachia* may play a role in impairing this process in *Cx. quinquefasciatus* infected with WNV.

Salivary gland barriers are another critical aspect for vector competence [61, 62]. In our study, although the transmission rate was higher for *Wolbachia*-free mosquitoes, the difference was not significant. These results suggest that the native *Wolbachia* protective effect did not occur at the salivary gland level, and that differences in transmission rate likely resulted from varying levels of WNV dissemination from the mosquito midgut.

Previous studies showed that antiviral protection conferred by native *Wolbachia* is correlated with its density in host tissues [63–65]. We found no significant changes in *Wolbachia* density between the initial F1 generation and the F7 generation used in artificial infection. These results suggest that the protection provided by native *Wolbachia* in the mosquitoes in our study was not attributable to variations in the endosymbiont density within host tissues.

How *Wolbachia* impairs viral replication in mosquitoes is unclear. Two mechanisms have been suggested. In the first, *Wolbachia* may compete with the virus for cellular resources, such as cholesterol, which is necessary for virus replication [66–69]. The second proposed mechanism is that the symbiont triggers the activation of the host's immune system, including the Toll, IMD and Jak/STAT signaling pathways, and antimicrobial peptides [16, 70–72]. *Wolbachia* appears to be inefficient in activating the immune system in mosquitoes where it naturally resides, such as *Cx. quinquefasciatus*, a phenomenon attributed to the extended period of co-evolution between the endosymbiont and the host [73–75]. Therefore, it is reasonable to hypothesize that inhibition of WNV by *Cx. quinquefasciatus* native *Wolbachia* may be primarily due to nutrient competition rather than be immune related. Supporting this hypothesis are studies that found no differences in the regulation of immune genes in *Culex* and *Aedes* mosquitoes due to their native *Wolbachia* [10, 74]. Further investigations on this mosquito species are warranted to validate this hypothesis.

## Conclusion

Our findings showed that *Cx. quinquefasciatus* from Cape Verde transmitted the tested L1 strain of WNV, highlighting the significance of continued surveillance efforts to prevent virus transmission within the country. Regarding the effect of the mosquito species native *Wolbachia* on WNV transmission, our results showed that when the endosymbiont was removed from Cx. *quinquefasciatus*, the mosquitoes exhibited higher WNV body titers compared to that in their counterparts carrying *Wolbachia*, which resulted in earlier dissemination but not transmission of WNV. Additional research is needed to elucidate the mechanisms underlying *Wolbachia*-mediated viral protection in natural hosts and the extent to which this protection may affect the epidemiology of WNV.

## Supplementary Information

Additional file 1: Table S1. Primer sequences employed in the molecular identification of the *Culex pipiens* complex species collected in Santiago Island, Cape Verde**.** Table S2. Primer sequences used to detect and quantify *Wolbachia* in *Culex quinquefasciatus* from Santiago Island, Cape Verde. Table S3. Probe and primer sequences used for West Nile virus detection by RT-qPCR.

## Data Availability

No datasets were generated or analyzed during the current study.

## Abbreviations

BSL3: Biosafety Level 3
CTAB: Cetyltrimethylammonium bromide
dpi: Days post-infection
PFU: Plaque-forming units
RT-qPCR: Quantitative real-time PCR
VIASEF: In vivo Arthropod Security Facility
WNV: West Nile virus
*wsp*: *Wolbachia* surface protein gene
