# Supplementary material for: Vector competence of Culex quinquefasciatus from Santiago Island, Cape Verde, to West Nile Virus: exploring the potential effect of the vector native Wolbachia
Source: Parasit Vectors. 2024 Dec 23;17:536. doi: 10.1186/s13071-024-06609-7 (PMC11668078; doi:10.1186/s13071-024-06609-7)
Supplement: Supplementary file 1 — Additional file 1: Table S1. Primer sequences employed in the molecular identification of the Culex pipiens complex species collected in Santiago Island, Cape Verde. Table S2. Primer sequences used to detect and quantify Wolbachia in Culex quinquefasciatus from Santiago Island, Cape Verde. Table S3. Probe and primer sequences used for West Nile virus detection by RT-qPCR. [file 13071_2024_6609_MOESM1_ESM.docx]

**Supplementary materials**

Table S1- Primer sequences employed in the molecular identification of Culex pipiens complex species collected in Santiago Island, Cape Verde.

| Species | Primers | Sequences (5’- 3’) | References |
| --- | --- | --- | --- |
| *Culex pipiens* complex | ACEquin | CCTTCTTGAATGGCTGTGGCA | [1] |
|  | ACEpip | GGAAACAACGACGTATGTACT |  |
|  | B1246s | TGGAGCCTCCTCTTCACGG |  |
|  |  |  |  |
| Other species (COI) | LCOI1490_F1 | GGTCAACAAATCATAAAGATATTG | [2] |
|  | HCOI2198_R1 | TAAACTTCAGGGTGACCAAAAAATCA |  |

Table S2- Primer sequences used to detect and quantify Wolbachia in Culex quinquefasciatus from Santiago Island, Cape Verde.

| Target | Primers sequences (5'-3') | Size (bp) | References |
| --- | --- | --- | --- |
| *wsp* (conventional PCR) | 81F: TGGTCCAATAAGTGATGAAGAAA | 610 | [3] |
|  | 691R: AAAAATTAAACGCTACTCCA |  |  |
|  |  |  |  |
| *wsp* (qPCR) | Forward: GCAAACAGTGTGGCAGCATT  Reverse: CACCAACACCAACACCAACG | 100 | [This study] |
|  |  |  |  |
| *18S* ribossomal | Cx18S-F: TCAGATGTTGATACCGTCGGC | 94 | [This study] |
|  | Cx18S-R: ATCAGGTCACACTACACCGC |  |  |

Table S3- Primers and probes used for West Nile Virus detection by RT-qPCR.

| Target | Primers sequences (5'-3') | Size (bp) | References |
| --- | --- | --- | --- |
| WNV Env | WNENV-F: TCAGCGATCTCTCCACCAAAG | 70bp | [4] |
|  | WNENV-R: GGGTCAGCACGTTTGTCATTG |  |  |
|  |  |  |  |
| Probe | FAM 5´-TGCCCGACCATGGGAGAAGCTC-3´ TAMRA | - |  |
|  |  |  |  |

**References:**

1. Smith JL, Fonseca DM. Rapid assays for identification of members of the *Culex* (*Culex*) *pipiens* complex, their hybrids, and other sibling species (Diptera: Culicidae). Am J Trop Med Hyg. 2004;

2. Folmer O, Black M, Hoeh W, Lutz R, Vrijenhoek R. DNA primers for amplification of mitochondrial cytochrome c oxidase subunit I from diverse metazoan invertebrates. Mol Mar Biol Biotechnol. 1994;3:294–9.

3. Zhou W, Rousset F, O’Neill S. Phylogeny and PCR-based classification of *Wolbachia* strains using wsp gene sequences. Proc R Soc B Biol Sci. 1998;265:509–15.

4. Lanciotti RS, Kerst AJ, Nasci RS, Godsey MS, Mitchell CJ, Savage HM, et al. Rapid Detection of West Nile Virus from Human Clinical Specimens, Field-Collected Mosquitoes, and Avian Samples by a TaqMan Reverse Transcriptase-PCR Assay. J Clin Microbiol. 2000;38:4066–71.
